# Advancing the Role of Neuroimmunity and Genetic Susceptibility in Gulf War Illness

**DOI:** 10.1016/j.ebiom.2017.11.021

**Published:** 2017-11-22

**Authors:** James P. O'Callaghan, Lindsay T. Michalovicz, Julie V. Miller, Kimberly A. Kelly

**Affiliations:** Centers for Disease Control and Prevention, National Institute for Occupational Safety and Health, Health Effects Laboratory Division, Morgantown, WV, United States

Gulf War Illness (GWI) is a chronic multi-symptom illness that has affected veterans of the 1991 Persian Gulf War for over two decades. Recently, research into GWI has greatly expanded, including investigations into potential initiating stimuli and conditions, current pathobiology, and promising treatments for this population of ill veterans. As the field of GWI research grows, it is important for researchers to further characterize and expand upon prior findings in order to bring the field closer to a comprehensive understanding of GWI and to develop therapies that treat the illness itself, not just its symptoms.

While the cause (s) of GWI remain largely unknown, most research supports a role for chemical exposures in theater (White et al., 2015) that initiate a protracted, largely neuroimmune-based disorder. Furthermore, the observation that a subset of veterans developed GWI, while nearly all soldiers were likely exposed to some combination of toxicants in theater, strongly supports the hypothesis that veterans with GWI may harbor some specific genetic-susceptibility. In the most recent publication from the Georgopoulos group, James et al. (2017) expand upon several of their previous studies verifying the protective role of HLA alleles related to brain function (Georgopoulos et al., 2015, James et al., 2016) in the observed subcortical brain atrophy associated with GWI (Christova et al., 2017). Here, James et al.’s (2017) evaluation of subcortical brain volumes in Gulf War veterans supported the suspected protective effect of the HLA class II allele DRB1*13:02 by finding a significantly higher subcortical volume in carriers of this allele. A hypothesis is presented that GWI is the result of persistent antigenicity resulting from the presentation of “novel” brain antigens following toxicant exposure. For example, exposure to acetylcholinesterase (AChE) inhibiting organophosphate compounds is well-studied as a potential initiator for GWI. In these models, there is the potential for either irreversibly phosphorylated, or “aged,” AChE or, as recently presented by Locker et al. (2017), the persistent “organophosphorylation” of neuroimmune-related targets to serve as a persistent antigen. Additionally, the presence of “auto-antibodies” against several neural proteins in the sera of veterans with GWI (Abou-Donia et al., 2017) suggests that changes in the brain following in-theater exposures has resulted in the release of brain antigens that have stimulated an immune response. Moreover, the extreme stress experienced in theater, which has been demonstrated to enhance the acute neuroinflammatory response to chemical toxicants (O'Callaghan et al., 2015, Locker et al., 2017), has the potential to perpetuate a persistent immune hypersensitivity to any potential GWI-related antigen (see Dhabhar and McEwen, 1996). The work presented in James et al. (2017) links these GWI studies to a potential mechanism involving genetic susceptibility based upon deficient antigen presentation facilitated by HLA class II allele genotype.

Ultimately, the culmination of all research into GWI should aid in bringing the field closer to effective treatment of this long-standing, chronic illness. As noted by James et al. (2017), the particular HLA alleles studied are frequently associated with chronic immune disorders, such as rheumatoid arthritis (RA), lupus erythematosus and multiple sclerosis (MS). Again connecting and expanding upon their previous work, Georgopoulos et al. (2017) identified similarities in HLA-related neural interactions between GWI, RA and MS. Not only does this further support the identification of GWI as a neuroimmune disorder, but the association of GWI with other well-studied immune/neuroimmune disorders provides the opportunity to implement treatment strategies already proven effective in related immune-based disorders. Indeed, computational modeling aimed at identifying FDA-approved pharmaceuticals that may “reset” the neuroimmune disruption present in GWI, has identified several drugs prescribed for chronic immune-related illnesses as potential treatments for GWI (Craddock et al., 2015).

While the “persistent antigen” model introduced by James et al. is attractive in that it provides a basis for the persistent symptoms of GWI, no pathogen has yet to be identified or implicated in this long-term illness. Of course, a latent pathogen may be the source, one that periodically emerges with conditions that ill veterans have been exposed to over the decades ensuing since the war. Physiological stressors and immune challenges (biologicals, inflammagens, chemical sensitizers) are potential candidates, but finding the culprits will remain a challenge that must be surmounted if we are to end the years of suffering caused by events that took place long ago in the Gulf War theater.

## Disclosure

The authors declared no conflicts of interest.
